# Optimizing the geometry of aerodynamic lens injectors for single-particle coherent diffractive imaging of gold nanoparticles

**DOI:** 10.1107/S1600576721009973

**Published:** 2021-11-16

**Authors:** Lena Worbs, Nils Roth, Jannik Lübke, Armando D. Estillore, P. Lourdu Xavier, Amit K. Samanta, Jochen Küpper

**Affiliations:** a Center for Free-Electron Laser Science, Deutsches Elektronen-Synchrotron DESY, Notkestrasse 85, 22607 Hamburg, Germany; b Department of Physics, Universität Hamburg, Luruper Chaussee 149, 22761 Hamburg, Germany; c Center for Ultrafast Imaging, Universität Hamburg, Luruper Chaussee 149, 22761 Hamburg, Germany; d Max Planck Institute for the Structure and Dynamics of Matter, Luruper Chaussee 149, 22761 Hamburg, Germany

**Keywords:** injectors, single particles, sample delivery, X-ray free-electron lasers, XFELs, nanoparticles, coherent diffractive imaging, numerical simulations, high-density beams

## Abstract

Single-particle X-ray diffractive imaging (SPI) of small (bio-)nanoparticles (NPs) requires optimized injectors to collect sufficient diffraction patterns to allow for the reconstruction of the NP structure with high resolution. Typically, aerodynamic lens-stack injectors are used for NP injection. However, current injectors were developed for larger NPs (>100 nm), and their ability to generate high-density NP beams suffers with decreasing NP size. Here, an aerodynamic lens-stack injector with variable geometry and a geometry-optimization procedure are presented. The optimization for 50 nm gold-NP (AuNP) injection using a numerical-simulation infrastructure capable of calculating the carrier-gas flow and the particle trajectories through the injector is also introduced. The simulations were experimentally validated using spherical AuNPs and sucrose NPs. In addition, the optimized injector was compared with the standard-installation ‘Uppsala injector’ for AuNPs. Results for these heavy particles showed a shift in the particle-beam focus position rather than a change in beam size, which results in a lower gas background for the optimized injector. Optimized aerodynamic lens-stack injectors will allow one to increase NP beam density, reduce the gas background, discover the limits of current injectors and contribute to structure determination of small NPs using SPI.

## Abstract 2

An optimization procedure of an aerodynamic lens injector with variable geometry is presented. The simulation results are validated by performing experiments on gold and sucrose nanoparticles. This work is envisioned to be an important step towards high-resolution single-particle imaging.

## 1. Introduction

Simulations have predicted the possibility of deriving high-resolution structures of biological macromolecules using X-ray free-electron lasers (XFELs) (Neutze *et al.*, 2000 ▸). The ultra-short and extremely bright pulses of coherent X-rays provided by free-electron lasers can outrun radiation-damage processes before the particle has time to structurally respond and eventually be destroyed by the deposited energy (Chapman *et al.*, 2006 ▸). Thus, the single-particle diffractive imaging (SPI) method at XFELs can be used to elucidate the structure of biological molecules (Bogan *et al.*, 2008 ▸; Aquila *et al.*, 2015 ▸) without the need for a highly ordered crystalline sample. SPI allows one to retrieve the 3D structure of biomolecules by reconstruction from a large number of 2D diffraction patterns assembled into a 3D diffraction volume, requiring high probability of an X-ray pulse interacting with an injected particle (Ekeberg *et al.*, 2015 ▸; Rose *et al.*, 2018 ▸; Lundholm *et al.*, 2018 ▸; Ayyer *et al.*, 2021 ▸). High-density particle beams with ideally one particle per pulse and focus volume are generated to use both X-rays and sample efficiently. However, for the atomic-resolution (∼100 pm) reconstruction of a protein, 10^5^–10^6^ diffraction patterns need to be collected (Poudyal *et al.*, 2020 ▸).

Delivery of high-density single-particle beams has been demonstrated using aerodynamic lens stacks (ALSs) to generate focused beams of aerosolized particles from ambient conditions into vacuum (Liu *et al.*, 1995 ▸; Bogan *et al.*, 2008 ▸). An ALS contains sets of thin apertures to manipulate the particles’ lateral spatial distribution before it exits through the last aperture into vacuum. Aerodynamic lenses enable successive contractions of a flowing particle beam and provide focusing to high particle densities for wide range of particle sizes (Benner *et al.*, 2008 ▸; Bogan *et al.*, 2010 ▸). Before adaption for SPI, they were mainly used in aerosol mass spectrometry to ensure a high transmission for a large particle-size range (Canagaratna *et al.*, 2007 ▸). A widely used injection system for SPI is the ‘Uppsala injector’, which usually contains an ALS (TSI AFL100). The Uppsala injector can deliver collimated or focused beams for a range of particle sizes, *e.g.* 0.1–3 µm (Hantke *et al.*, 2014 ▸). It was successfully used in various experiments at XFEL facilities and allowed injection of 30 nm to 1 µm particles (Seibert *et al.*, 2011 ▸; Hantke *et al.*, 2014 ▸; van der Schot *et al.*, 2015 ▸; Bielecki *et al.*, 2019 ▸; Ho *et al.*, 2020 ▸). A recent experiment performed at EuXFEL demonstrated the successful collection of more than 10 000 000 diffraction patterns from single gold nanoparticles (AuNPs) using this injector and shows the opportunities provided by careful sample preparation and injection (Ayyer *et al.*, 2021 ▸).

**Figure 1:**
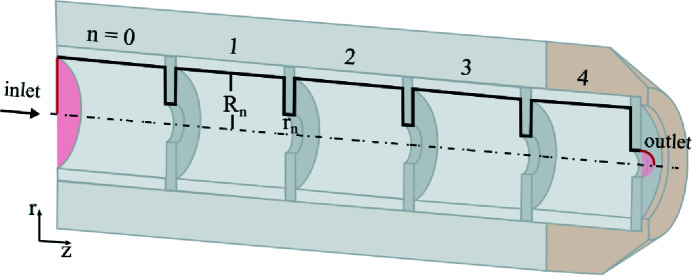
A schematic of the ALS geometry, which is cylindrically symmetric about the dashed line. Carrier gas flows from left to right. The black solid line is the 2D geometry used for the simulations, consisting of five aperture and tube pieces. The inner radius (*R*_*n*_) of the tube as well as the lens-aperture radius (*r*_*n*_) can be changed individually (Roth *et al.*, 2021 ▸); see the main text for details.

However, currently, sample injection and beam formation are the bottleneck of collecting large data sets of small-bio-particle diffraction patterns, and injection schemes have to be modified accordingly (Bielecki *et al.*, 2020 ▸). The geometry of the AFL100 is fixed, typically such that it can deliver particle beams for a broad size range. The remaining parameter for tunability of the particle-beam’s focus size during an experiment is the inlet pressure before the ALS (Hantke *et al.*, 2018 ▸). To circumvent the increase of inlet pressure to generate a smaller particle-beam focus and thus an increase of pressure in the experimental chamber, we have designed and used a new particle injector with variable geometry, as shown in Fig. 1 ▸, *i.e.* the inner tube diameter and the aperture diameter can be changed (Roth *et al.*, 2018 ▸, 2021 ▸) to produce the highest particle-beam density for a given particle size. In addition, the speed of the particles is important in SPI experiments. It should be as slow as possible to increase the particle-beam density and thus hit rate, but with increasing repetition rates at XFEL facilities the particle speed has to be sufficiently fast to avoid interaction of NPs with two X-ray pulses. For the full repetition rate of 4.5 MHz at EuXFEL (Decking *et al.*, 2020 ▸) and an X-ray focus size of 2 µm, the particle speed has to exceed 10 ms^−1^ to enter the interaction region without interacting with the previous pulse, which can damage or scatter off the sample.

Here, we present the geometry optimization for aerosolized spherical AuNPs of 50 nm diameter at typical inlet conditions for SPI experiments, based on the generated particle-beam properties. The numerical-simulation infrastructure used is presented elsewhere (Roth *et al.*, 2018 ▸; Welker *et al.*, 2022 ▸). To validate our simulation results, we compare them with experimental data for both AuNPs and sucrose spheres. AuNPs, when synthesized and prepared well, show a narrow size distribution similar to bio-particles and are therefore good benchmark samples for sizing and focusing experiments. AuNPs have a high scattering power that results in high detection efficiencies both in in-laboratory detection methods (Awel *et al.*, 2016 ▸; Worbs *et al.*, 2019 ▸) and in X-ray diffractive imaging, making them useful for benchmarking data analysis and structure-determination methods (Ayyer *et al.*, 2021 ▸). Furthermore, AuNPs exhibit distinct physical and chemical properties with potential applications ranging from quantum electronics to biomedicine and potential drug-delivery systems (Schmid & Simon, 2005 ▸; Dykman & Khlebtsov, 2012 ▸). Sucrose particles are often used at XFEL facilities for alignment in commissioning and startup experiments (Bielecki *et al.*, 2019 ▸; Ho *et al.*, 2020 ▸), as the number density of the generated sucrose spheres is high and the particle beam can be observed easily while aligning the injector to the X-ray beam. Most importantly, the mass density of sucrose NPs, and thus their focusing behavior, is comparable to that of biological matter, rendering them a good prototypical benchmark system for bio-NPs.

## 2. Methods

### 2.1. Geometry optimization

Simulations of the ALS were performed as follows. First, we calculated the flow field of the carrier gas inside a given 2D cylindrically symmetric geometry using a finite-element solver for the Navier–Stokes equations (COMSOL, 2019 ▸). The flow field was calculated within the ALS geometry and extended after the exit with a quarter circle with the radius of the last aperture serving as the gas-expansion region of the vacuum chamber, as shown in Fig. 1 ▸. The carrier gas was assumed to be nitrogen, as the particles were aerosolized using electrospray ionization, where the used gas mixture consists of ∼90% nitrogen and ∼10% CO_2_. As boundary conditions for the flow field, we used a mass-flow conservation of 13 mg min^−1^ as the inlet condition and a pressure of 10^−4^ mbar (1 mbar = 100 Pa) at the end of the flow field along the semi-circle. These values were experimentally used in a previous SPI experiment (Roth *et al.*, 2021 ▸). The limit of the mass-flow inlet is given by the pressure limits in the interaction chamber of SPI endstations, which typically require *p* < 10^−5^–10^−4^ mbar depending on the gas type. Additional flow-field calculations were performed using the inlet pressure as a boundary condition. Second, the trajectories of 100 000 particles for a given flow field were calculated with an in-house Python particle-tracing code (Roth *et al.*, 2018 ▸): see also the meanwhile publicly available code (Welker *et al.*, 2022 ▸). This particle-tracing code assumes spherical particles. As most small bio-particles are non-spherical, future particle-tracing codes will have to take the particle shape into account (Wang & McMurry, 2006*b* ▸). Particles were introduced into the flow field with a uniform radial distribution covering the diameter of the first tube piece. We assumed the particles’ velocity to be equal to the flow-field values. We simulated trajectories of 50 nm diameter spherical particles with a density of 19.32 g cm^−3^, corresponding to the bulk density of gold. Transmitted particles were propagated further with their terminal speeds at the border of the flow field. Then, we determined the width of the resulting particle beam depending on the distance from the ALS exit, *i.e.* the last aperture. Beam widths *d*_70_ were specified as the diameter that encompasses 70% of the particles; *d*_70_ is a useful and robust metric as it is independent of the actual beam shape. Nevertheless, outside of the ALS, all simulated particle beams showed a peak-like radial distribution with the maximum of the particle density in the center (*r* = 0 mm).

The ALS consists of *n* = 5 aperture/tube pieces stacked onto one another (see Fig. 1 ▸). The lens aperture radius *r*_*n*_ and the inner tube radius *R*_*n*_ can easily be adjusted. In our ALS, the aperture radius can be chosen from 0.75 to 5 mm in 0.25 mm steps. The lens apertures are interchangeable. The inner tube diameter could have been chosen from parts with radius 2, 3, 4, 5, 6, 7.5, 8 or 10 mm, which were available in stock; in principle, any size would be possible. The inner diameter of the tubes is adjusted by adding an additional tube into the standard 10 mm diameter pieces. The length of the tubes would be a possible optimization parameter, which was fixed in the current study to avoid the added dimensions and complexity in the fit.

As the variety corresponds to more than 7 × 10^10^ combinations, we approached the optimization as follows. Our optimization procedure was performed iteratively from the exit to the entrance of the ALS, as the last aperture radius (*r*_4_) largely determines the focus position and size of the particle beam (De La Mora & Riesco-Chueca, 1988 ▸). We started the optimization in the last piece of the ALS, *r*_4_ and *R*_4_. The particles were introduced into the flow field with a uniform radial distribution covering *r*_initial_ = 0.02 mm, mimicking the situation where the preceding lenses have already prefocused the particle beam. The initial particle velocity was set equal to the flow-field speed. The best *r*_4_ and *R*_4_ combination fulfills the following conditions: the transmission was >90%, the focus was at *z* > 4 mm to suppress background scattering from the housing of the ALS, and it resulted in the smallest beam diameter. With this optimized *r*_4_ and *R*_4_ combination, we then optimized *r*_3_ and *R*_3_ for the same requirements of the particle beam, and this was subsequently iterated for all lenses with increasing initial radial distribution of the particles, *i.e.* 0.02 mm for pieces 4 and 3, 0.5 mm for piece 2, 1 mm for piece 1, and the whole radius of the lens filled before the first aperture. This optimization procedure reduces the efforts to 160 combinations per lens and <1000 overall.

In addition, we performed simulations using the ‘lens calculator’ (Wang & McMurry, 2006*a* ▸) with similar input values for particle and flow specifications to compare the result with our optimized geometry. Details on these simulations are given in the supporting information.

### 2.2. Experimental setup

**Figure 2:**
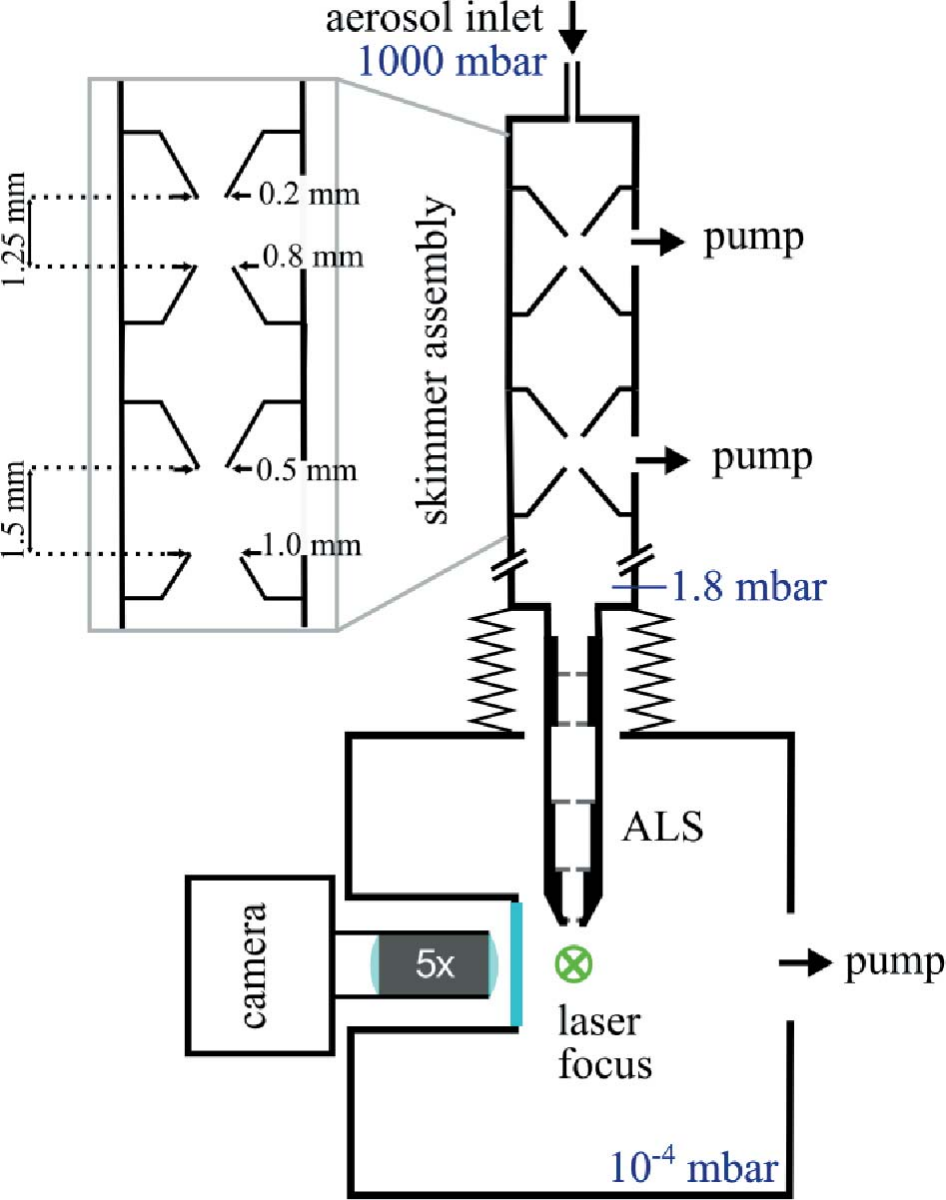
A schematic of the experimental setup for the characterization of NP beams. The aerosol passed a skimmer assembly to remove most of the carrier gas, and the particles were focused using an ALS and entered the main vacuum chamber, where the particle beam was crossed by a laser beam. The light scattered off the particles was collected using a camera-based microscope system (Awel *et al.*, 2016 ▸; Worbs *et al.*, 2019 ▸).

We measured the particle-beam evolution of AuNPs from the optimized ALS geometry. The schematic of our experimental setup is shown in Fig. 2 ▸. It consists of four main parts: an aerosolization chamber, a differentially pumped transport tube, the ALS system for particle-beam formation and the detection region for visualization of the particle beam. To generate isolated test particles from the liquid sample, we injected spherical AuNPs with a diameter of 27 ± 2.25 nm into 5 m*M* of ammonium acetate (AmAc) with a concentration of 10^11^ particles ml^−1^ and a 2% sucrose solution into 20 m*M* of AmAc using a commercial electrospray (TSI Advanced Electrospray 3482). The aerosolized NPs passed through a differentially pumped skimmer assembly for pressure reduction. The particles were focused into the detection chamber using the ALS. The pressure above the entrance of the ALS was 1.8 mbar (Pfeiffer Vacuum CMR 361). In the main chamber, the pressure was kept at 2.5 × 10^−4^ mbar. The ALS is mounted on a motorized *xyz* manipulator to perform height scans and measure the particle-beam evolution.

Particles were detected using a side-view illumination scheme (Awel *et al.*, 2016 ▸). An Nd:YAG laser (Innolas SpitLight, 532 nm, pulse duration of 11.5 ns, pulse energy up to 240 mJ at 532 nm, 20 Hz repetition rate) was focused into the center of the vacuum chamber intersecting the particle beam. The light scattered off the particles was collected using a camera-based microscope system (Awel *et al.*, 2016 ▸; Worbs *et al.*, 2019 ▸) consisting of a long-working-distance objective (Edmund Optics, 5× magnification, numerical aperture of *N*_a_ = 0.14, working distance of *d* = 34 mm, depth of field of 14 µm) and a high-efficiency sCMOS camera (Photometrics PrimeB95, quantum efficiency of 0.95 at 532 nm, 1200 × 1200 pixels). This microscope yields a nominal resolution of 0.54 pixel µm^−1^. Images were collected with a 1 ms exposure time synchronized to the laser at 20 Hz such that every frame covered one laser pulse. For every distance of the ALS and the laser, we recorded 10 000 images for the AuNP sample and 2000 images for the sucrose sample. We determined the positions of the particles by analyzing the images using a centroiding algorithm based on Hessian blob finding (Marsh *et al.*, 2018 ▸). The particles’ positions were converted into a 2D histogram (see the supporting information for details). The width of the particle beam is determined from the projection of the particle beam onto the laser-propagation axis. The beam diameter is shown as *d*_70_.

## 3. Results

### 3.1. Optimization and simulation results

**Figure 3:**
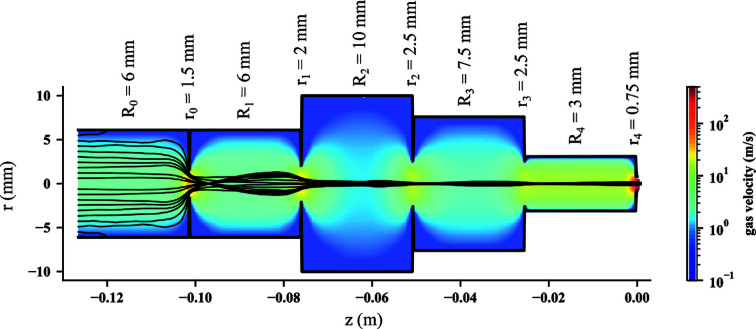
The optimized ALS geometry. Tube and aperture radii are specified above the device and the corresponding nitrogen-gas flow field for the injection of 50 nm AuNPs at 13 mg min^−1^ mass flow is depicted in false color. Representative (calculated) particle trajectories are shown by black lines, with gas- and particle-flow direction from left to right. A clear focusing effect of the different parts of the ALS can be observed through the radial narrowing of the set of particle trajectories.

The optimization process resulted in one final geometry which produced a particle beam to our specifications. The resulting optimized-lens-stack geometry is shown in Fig. 3 ▸. From entrance to exit, the lens-tube and aperture radii are first increasing, then decreasing. The smallest lens-tube radius and aperture radius are obtained for the last lens piece. Values are given next to the geometry. The velocity-flow field for 13 mg min^−1^ mass flow and particle trajectories for 50 nm AuNPs at different inlet positions (black solid lines) are shown in Fig. 3 ▸, demonstrating a clear focusing effect of the ALS.

For this optimized injector, we calculated the Stokes numbers for all the apertures and tubes. They are very close to the optimal Stokes number for the pressure range that we are working in (see Table S2 of the supporting information for details).

**Figure 4:**
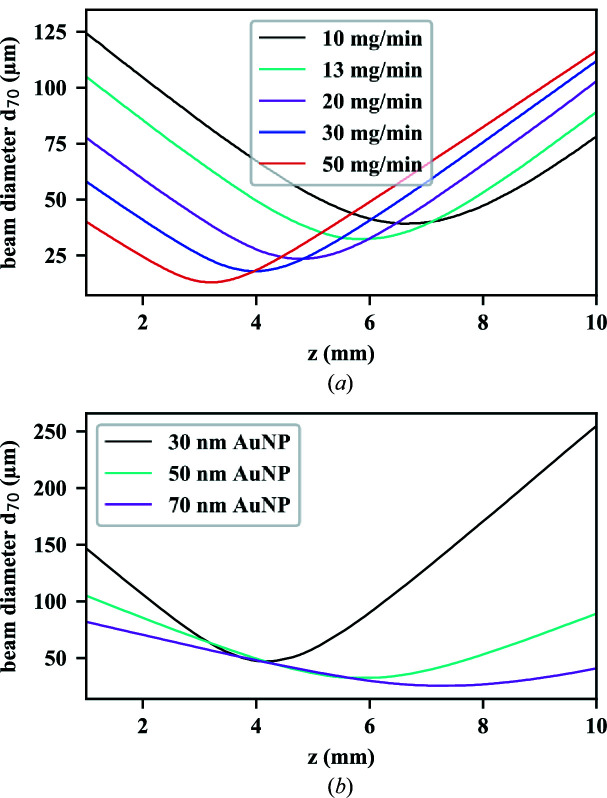
(*a*) Particle-beam evolution curves of the optimized injector for 50 nm AuNPs at different gas-mass flows. The width of the particle beam was determined as *d*_70_. With increasing mass flow and thus pressure before the ALS, the particle-beam focus becomes harder, *i.e.* it moves closer to the ALS exit and gets smaller. (*b*) Particle-beam evolution curves of the optimized injector for different AuNP sizes at 13 mg min^−1^ mass flow. With increasing particle size, the particle-beam focus decreases and moves further away from the ALS. The convergence increases with decreasing particle size.

For this ALS geometry, the 50 nm AuNP beam was focused at a distance of 5.8 mm from the ALS exit with a particle-beam width of 33 µm (*d*_70_). The particle-beam evolution for 13 mg min^−1^ mass flow is shown in Fig. 4 ▸(*a*) as the cyan curve. The particle-beam evolution is shown as the beam width (*d*_70_) depending on the distance *z* from the ALS exit. We simulated the focusing behavior for different mass-flow conditions between 10 and 50 mg min^−1^. With increasing mass flow, the focus shifted closer to the exit of the ALS and the focus size decreased. At 50 mg min^−1^ mass flow, the particle-beam focus size decreased to 13 µm at a distance of 3.2 mm. Similar behavior has been shown experimentally for the Uppsala injector (Hantke *et al.*, 2018 ▸). Therefore, working at higher mass flow is desired, but it will increase the amount of gas introduced into the interaction chamber and result in a higher pressure and thus a higher gas-scattering background in diffractive imaging experiments.

At 13 mg min^−1^ mass flow the AuNPs exiting from the optimized ALS had a mean speed of 29 ms^−1^. Mean speeds and beam-diameter values for different flow conditions are given in the supporting information. The behavior of the ALS optimized for 50 nm AuNPs was compared with smaller and larger diameters of the AuNPs, as shown in Fig. 4 ▸(*b*). For smaller AuNPs the focus moved closer to the ALS and was larger, whereas bigger particles were focused further away and showed a smaller focus size. An interesting feature observed was the change of the convergence depending on the particle size: the smaller the particles were, the larger the convergence became. A precise positioning for small particles becomes necessary to meet the particle-beam focus. This change of the convergence is due to the larger momentum of larger particles interacting with the gas-flow field.

The optimized geometry obtained using the lens calculator (Wang & McMurry, 2006*a* ▸) is shown in Fig. S3 of the supporting information. All aperture and tube radii were larger than in our geometry. This ALS produced a particle-beam width of 817 µm at 5 mm, *i.e.* it resulted in a much larger particle beam with correspondingly strongly reduced density compared with our fully optimized geometry.

### 3.2. Experimental results

**Figure 5:**
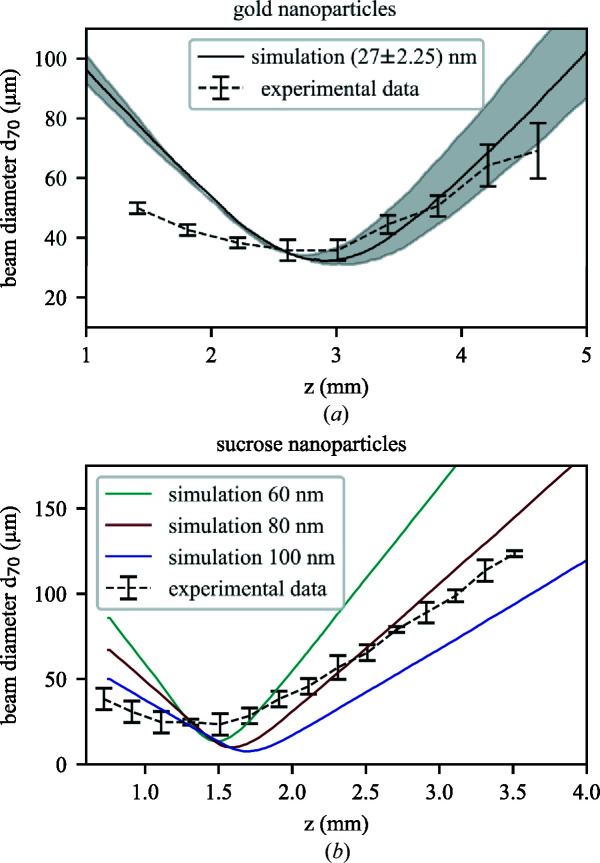
(*a*) Experimental particle-beam-size evolution for 27 ± 2.25 nm AuNPs (black dashed line). Simulated beam evolution is shown for 27 nm (black solid line) with a spread of the beam diameter due to the size distribution of ±2.25 nm (gray area). (*b*) Experimental particle-beam-size evolution for sucrose spheres (black). The experimental data agree reasonably well with a simulated particle size of 80 nm (dark red).

We measured particle-beam evolution curves of AuNPs and sucrose particles. The AuNP data with standard errors are shown in Fig. 5 ▸(*a*) as beam diameter (*d*_70_) depending on the distance from the injector exit, along with simulations for a particle size of 27 ± 2.25 nm using the experimentally measured inlet pressure of 1.8 mbar above the ALS. The experimental and simulated particle-beam diameters agree well, especially at and after the focus of the particle beam. Some deviations are observed before the focus, where the simulation overestimates the beam diameter, *e.g.* by a factor of ∼1.5 at *z* = 1.4 mm. However, the most relevant parameters for SPI experiments, the focus position and focus size, are in excellent agreement between experiment and simulation. The same experiment is repeated for a 2% sucrose solution to generate spherical sucrose particles in the electrospray process with a broad size distribution of ∼80 nm, shown in the supporting information. The sucrose particle-beam evolution is shown in Fig. 5 ▸(*b*) with standard errors (black) and compared with simulations for different sizes of sucrose spheres (ρ = 1.59 g cm^−3^). Overall, the experimental data are described well by the simulation for 80 nm sucrose spheres. Similar to the AuNP data, the simulation agrees well with our data after the focus, although before the focus the simulation deviates by a factor of ∼1.6 at *z* = 0.8 mm. This mismatch is partly due to the broad experimental size distribution, *i.e.* the experimental data do not correspond to a single particle-size simulation.

### 3.3. Uppsala-injector simulation

The Uppsala injector (AFL100) has been introduced before and used in various experiments at XFELs (Seibert *et al.*, 2011 ▸; Hantke *et al.*, 2014 ▸; Bielecki *et al.*, 2019 ▸; Ho *et al.*, 2020 ▸). We simulated its focusing of 50 nm AuNPs and compared it with our optimized ALS.

**Figure 6:**
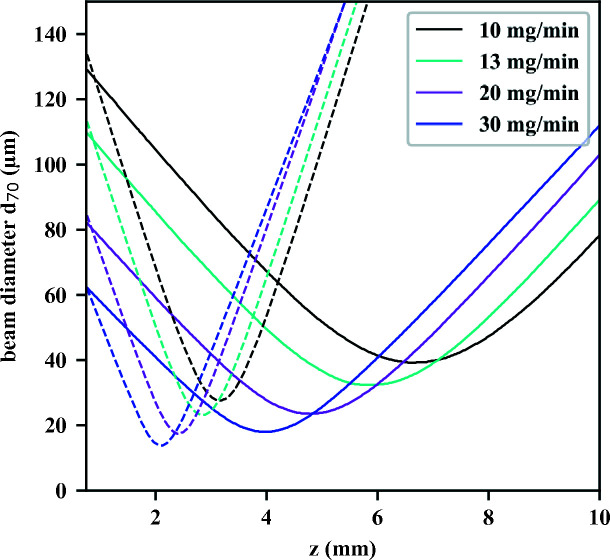
Simulated particle-beam evolution curves of 50 nm AuNPs exiting from the Uppsala injector (dashed lines) for different mass-flow conditions in comparison with the corresponding focusing curves from our optimized injector (solid lines).

The beam evolution curves for 50 nm AuNPs at different mass-flow conditions for this injector are shown in Fig. 6 ▸ (dashed lines), along with the focusing curves for our optimized injector (solid lines). The simulated transmission of 50 nm AuNPs through both injectors was above 90%, *i.e.* at 13 mg min^−1^ mass flow the transmission was 91.9% for the AFL100 and 93.4% for our optimized injector. At the same mass flow, the AFL100 showed slightly smaller mean speeds of the exiting particles than our optimized injector. As an example, at 13 mg min^−1^ mass flow, 50 nm AuNPs exiting the Uppsala injector showed a mean speed of 27 m s^−1^. In comparison, particles in our injector reached a speed of 29 m s^−1^. A detailed list of speeds depending on mass flow is shown in the supporting information.

## 4. Discussion

Our simulations show that the focus size is comparable for both injectors, but the difference is in the focus position and the convergence. Our injector focuses the particles further downstream and the focusing is not as hard as for the AFL100. Generating a focused particle beam further away from the injector exit has the advantage of a lower background from the nitrogen and CO_2_ gas. Light gas diverges fast from the exit of the ALS into the vacuum chamber.

The focus position of the AFL100 is closer to the injector exit and can cause problems when using smaller particles and particles with smaller density, such as bio-particles: the smaller and lighter the particles, the closer the focus position. As an example, simulations for 10 nm AuNPs at 13 mg min^−1^ nitrogen mass flow for the AFL100 showed that the focus position moves very close to the ALS exit, below *z* = 1.5 mm, and the transmission is reduced to 59%. Our 50 nm-optimized injector still shows a transmission of 79% for those particles and a focus position > *z* = 2 mm (see the supporting information for details). The same behavior holds for the particle density: the lower the particle density (biomolecules), the closer the particle-beam focus becomes. For isolated proteins, it is almost impossible to focus the particle beam with these injectors. In this case, an appropriate geometry optimization could result in a geometry that focused the particle beam further away from the injector exit. In addition, the particle transmission for a 10 nm bio-particle would decrease owing to lower density compared with the density of an AuNP, which is ∼20 times larger. Lower particle density increases Brownian motion for the same size and therefore decreases particle transmission though the ALS for bio-particles. This could be mitigated by lowering the temperature (Samanta *et al.*, 2020 ▸), if compatible with the envisioned study.

## 5. Conclusions

We have presented an optimization procedure of an ALS for 50 nm AuNPs using our previously developed computer-simulation framework for ALS injectors (Roth *et al.*, 2018 ▸; Welker *et al.*, 2022 ▸), including the results of this optimization. We experimentally benchmarked the optimized geometry for beams of spherical gold and sucrose NPs. Both particle-beam evolution curves are in good agreement with the simulations. This validates our simulation framework, which can be used to gain further insight into the fluid-dynamics focusing process and to develop optimized particle injectors for different sizes and materials, as well as for different experimental conditions, such as inlet pressure and gas type.

We compared our optimized injector with the widely used AFL100 Uppsala injector for 50 nm AuNPs. Both injectors create a focused particle beam for different inlet mass-flow conditions. The main difference is observed in the particle-beam focus position, which for our optimized injector is further downstream. This change reduces the carrier-gas background at the focus and will be greatly beneficial for X-ray diffractive imaging, especially of small bio-particles that exhibit only small scattering signals.

Our variable injector geometry allows us to vary the particle-beam focus independently from the inlet pressure by varying the geometry and thus keeping the pressure after the injector, *i.e.* in the X-ray interaction region, constant. Generating high-quality particle beams of NPs not only allows for structure determination by an increased number of collected diffraction patterns but also opens the field of time-resolved imaging of NP dynamics in future pump–probe-type experiments at XFELs.

## 6. Related literature

The following additional references are cited in the supporting information for this article: Horke *et al.* (2017) ▸; Yon *et al.* (2020) ▸. 

## Supplementary Material

Supporting information. DOI: 10.1107/S1600576721009973/te5083sup1.pdf
