# Supplementary material for: Optimizing the geometry of aerodynamic lens injectors for single-particle coherent diffractive imaging of gold nanoparticles
Source: J Appl Crystallogr. 2021 Nov 16;54(Pt 6):1730–7. doi: 10.1107/S1600576721009973 (PMC8662975; doi:10.1107/S1600576721009973)
Supplement: Supplementary file 1 [file j-54-01730-sup1.pdf]

JOURNAL OF
APPLIED
CRYSTALLOGRAPHY

Volume 54 (2021)

Supporting information for article:

Optimizing the geometry of aerodynamic lens injectors for single-particle coherent diffractive imaging of gold nanoparticles

Lena Worbs, Nils Roth, Jannik Lübke, Armando D. Estillore, P. Lourdu Xavier, Amit K. Samanta and Jochen Küpper

Supplementary Information: Optimizing the geometry of aerodynamic lens injectors for single-particle coherent diffractive imaging of gold nanoparticles

Lena Worbs,[1,2] Nils Roth,[1,2] Jannik Lübke,[1,2,3] Armando D. Estillore,[1]
P. Lourdu Xavier,[1,4] Amit K. Samanta,[1] and Jochen Küpper[1,2,3,*]

[1] *Center for Free-Electron Laser Science, Deutsches Elektronen-Synchrotron DESY, Notkestraße 85, 22607 Hamburg, Germany*
[2] *Department of Physics, Universität Hamburg, Luruper Chaussee 149, 22761 Hamburg, Germany*
[3] *Center for Ultrafast Imaging, Universität Hamburg, Luruper Chaussee 149, 22761 Hamburg, Germany*
[4] *Max Planck Institute for the Structure and Dynamics of Matter, Luruper Chaussee 149, 22761 Hamburg, Germany*
(Dated: 2021-09-17)

I. GAS-FLOW EFFECTS ON FOCUSING

We simulated the particle beam for 50 nm AuNP for the optimized injector and the "Uppsala-injector" (AFL100) for different nitrogen gas mass flow. The resulting values for the particle velocity and the focus size are shown in Table I. With increasing nitrogen mass flow, the mean velocity increased and the focus size decreased for both injectors. At the same mass flow, our optimized injector showed slightly larger mean velocities and slightly larger focus sizes, which does not make a significant difference.

II. FOCUSING 10 NM AUNPS

A principal and longstanding goal of single particle diffractive imaging is to image a single protein [1]. Toward this goal of smaller nanoparticle sizes, we simulated the focusing of 10 nm AuNP spheres by the AFL100 and our optimized ALS geometry, assuming a mass flow of 13 mg/min. The beam-size evolution is shown in Figure 1. We see the same behavior for larger particles: The beam focus size is comparable, 109 µm for the AFL100 and 115 µm for our injector, and also the speed of the particles is similar for the two injectors, 54±7 m/s compared to 56±5 m/s. The main difference occurs in the focus position, i. e., 1.13 mm and 2.13 mm, and the transmission. For the AFL100 only 59 % of the particles are transmitted through the whole ALS, whereas our injector transmission reaches 79 %. The focus position further away from the injector tip strongly reduces the background signal from gas scattering, i. e., in this case from 0.054 mbar to 0.01 mbar. The pressure map is shown in Figure 2. It shows the pressure drop after the exit aperture. For these calculations, we calculated the flow field in an extended region shaped as a cone up to 5 mm after the exit. Similar behavior of pressure drop after a convergent nozzle has been shown experimentally [2].

	optimized injector		AFL100	
mass flow (mg/min)	mean velocity (m/s)	focus size (µm)	mean velocity (m/s)	focus size (µm)
5	-	-	18	46.2
10	26	39.2	24	27.6
13	29	32.4	27	23.2
20	35	23.4	33	17.4
30	40	18.0	39	13.8
50	50	13.0	-	-

TABLE I. Detailed values from 50 nm AuNP simulations for the optimized lens and the "Uppsala-injector" (AFL100) for different mass flow inlet values.

* jochen.kuepper@cfel.de;
https://www.controlled-molecule-imaging.org

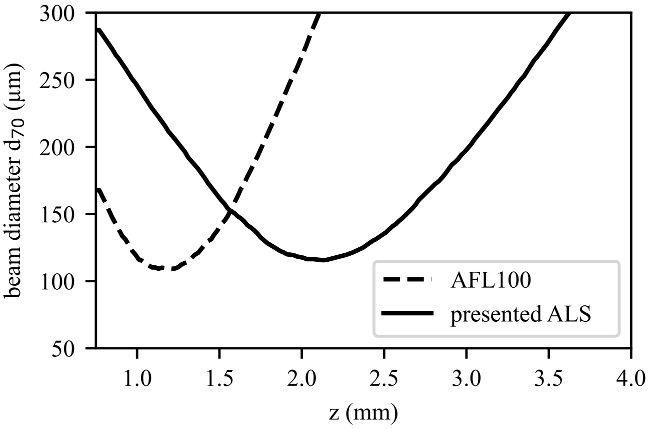

FIG. S1. Particle beam evolution for 10 nm AuNP spheres at 13 mg/min mass flow for the AFL100 (dashed line) and our 50 nm-AuNP-optimized injector (solid line).

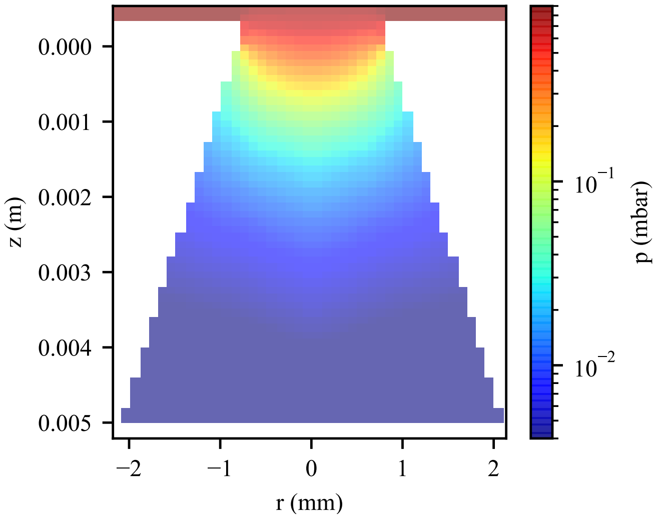

FIG. S2. Pressure map in an extended region after the ALS exit at 13 mg/min mass flow.

lens piece n	pressure (Pa)	Mach number	Reynolds number	Stokes number
0	102.4	0.12	8.4	1.36
1	98.7	0.07	6.3	0.59
2	97.2	0.05	5.0	0.31
3	96.5	0.05	5.2	0.31
4	94.5	0.65	20.5	11.77

TABLE II. Mach number, Reynolds number and Stokes numbers at the different lens pieces through the optimized injector. The values of the pressure were taken from the calculated flow fields.

III. OPTIMIZED GEOMETRY FLOW PARAMETER DISCUSSION

We calculated the Mach-number, Reynolds-number and Stokes number as defined in [3] for 50 nm AuNPs in our optimized geometry. The results are shown in Table II. The Stokes numbers are very close to the optimal Stokes number for the pressure range we are working in. In our geometry, the Stokes number at the first aperture r_0 is larger than the optimal Stokes number (≈ 1), resulting in the crossing of the center line of the particle trajectories [4] (see details in main manuscript). However, the main concern in crossing the center line and the diverging particle-beam is

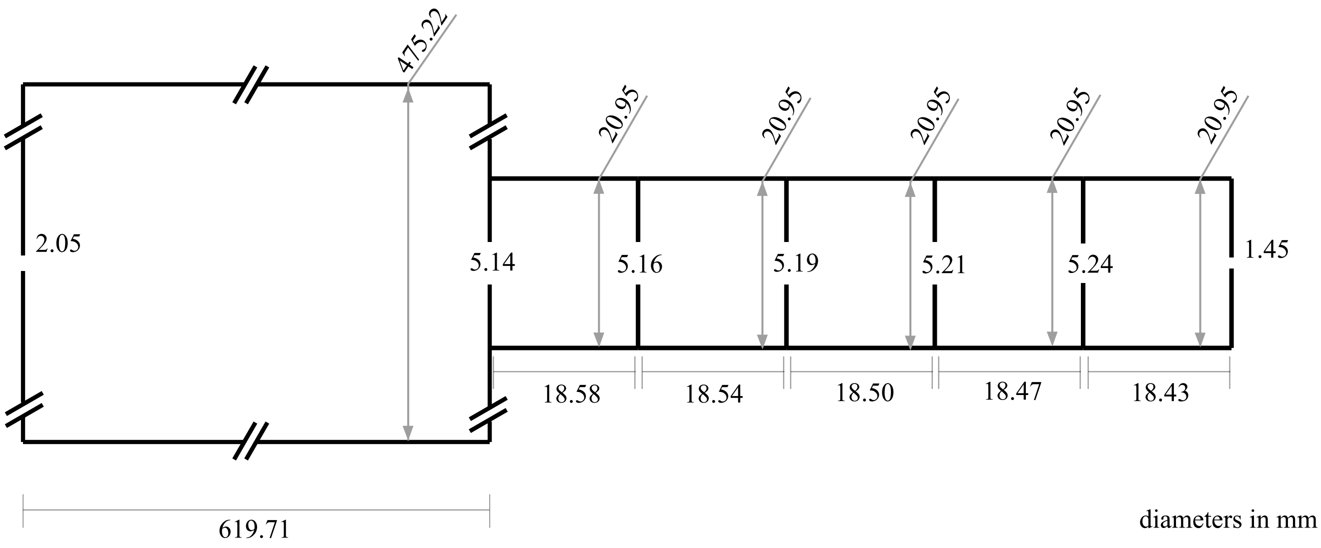

FIG. S3. Geometry of the aerodynamic lens system as a result of the lens calculator [3]. Particle and gas flow are from left to right. Values are given as diameters in mm.

particle loss, which is not observed in our case. All particles that are transmitted until the first aperture are transmitted though the whole lens geometry.

IV. LENS CALCULATOR RESULTS

We used the lens calculator [3] with input parameters of Nitrogen as the carrier gas at room-temperature, 115 Pa pressure before the inlet, so that the pressure in the relaxation chamber was comparable to the pressure in our injector before the first lens (103 Pa). The volumetric flow rate of 0.01 slpm corresponds to 13 mg/min in our optimization and the particle properties were set to a density of 19320 kg/m^3 and a diameter of 50 nm. The geometry from the lens calculator yielded a non-optimal particle-beam for the 50 nm AuNPs with a focus position inside the ALS. The generated particle-beam had a diameter after the exit of 798 µm and of 817 µm at 5 mm distance according to the output of the lens calculator. The geometry is shown in Figure 3 with the calculated diameters of the apertures, tubes and the length of the tubes next to it. In contrast to our optimized injector geometry, all tube diameters were equal [4]. The transmission of the geometry was given with 87 %.

In comparison, our optimized injector showed a higher transmission and a focused particle beam with a focus width of 33 µm.

The lens calculator is a useful tool to calculate the geometry quickly, but cannot be used to optimize the geometry based on the particle-beam properties.

V. SAMPLE PREPARATION

A. AuNPs

Citrate-capped spherical AuNPs (Sigma Aldrich) were ligand-exchanged, purified, and concentrated. As XFEL diffractive imaging is highly sensitive to the size of the particles interacting with the XFEL beam one needs to have highly-pure, salt- and impurity-layer-free target objects delivered to the interaction region. To mimic the XFEL experimental conditions in the lab-based optical-laser imaging, we used highly-concentrated AuNPs in a volatile buffer to get consistent size distributions with different flow-rates upon electrospray aerosolization, which was characterized using ES DMA-CPC (TSI, 3080 and 3786). Commercial samples as well as self-grown unpurified AuNP samples contain unwanted impurities and salts, which would impede the electrospray injection and not giving consistent size distributions with different flow rates. Citrate-capped AuNPs were ligand exchanged with bis(p-sulfonatophenyl)phenylphosphine dihydrate dipotassium salt (BSPP) (Sigma-Aldrich), to avoid aggregation at high concentration and different conditions

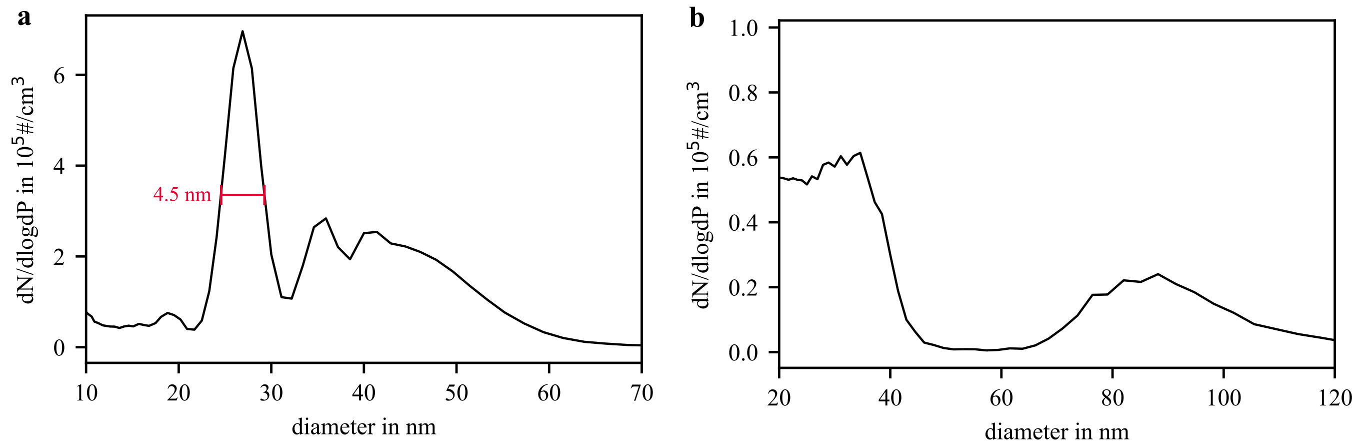

FIG. S4. DMA-CPC size distributions measured for (a) gold spheres and (b) 2 % sucrose solution. The conditions for the experiment are kept constant from the DMA measurements.

like repeated centrifugation during the purification [5]. Phosphine coating increases the overall negative charge of the particles and thus increases the stability of particles at a range of buffer conditions.

Briefly, 100 ml of AuNPs were incubated with 40 mg of of BSPP overnight, and then 5 M NaCl was added dropwise to the AuNP dispersion, until the color changed from red to dark blue. The blue dispersion was centrifuged at 1600 rcf for 30 min. The salty supernatant was discarded and the pellet was resuspended in 1 ml 2.5 mM BSPP and 1 ml methanol, centrifuged again (1600 rcf, 30 min), and resuspended in 1 ml 2.5 mM BSPP. The resulting AuNP solution was of burgundy-red color. Then the AuNP were centrifuged (Eppendorf) further 6 times at 2000 rcf for 15 min each, and redispersed in 250 µl Milli-Q water with 2.5 mM ammonium acetate. This process was optimized until the correct AuNP size was obtained for different flow-rates in the ES-DMA consistently as shown in Figure 4 a for the ES settings used during the experiment. A broad distribution at larger diameters from 30 to 60 nm was observed and ascribed to dimers generated in the ES process. We experimentally exclude larger (brighter) particle hits in our scattering experiment later.

UV-VIS-spectroscopic characterization (Nanodrop) of AuNPs after ligand exchange did not show indication of any aggregation and was consistent with the surface plasmon resonance (SPR) peak before ligand exchange and purification. The TEM analysis indicated the average core diameter of the AuNPs to be 24 nm, providing further indication that the larger particles detected in DMA-CPC were dimers created in the ES process. The mean size was calculated from TEM-based sizing for a sample size of 500 AuNPs, see Figure 5.

B. Sucrose

In addition, a 2 % sucrose solution was used to generate sucrose balls in the electrospray process. The size distribution of the generated sucrose balls is flow rate dependent. A DMA size distribution taken at the same electrospray settings as in the experiment is shown in Figure 4 b. We fitted a Gaussian distribution to the second peak. The peak particle size is 88 nm and the size distribution is rather broad with a FWHM of 29 nm. At this flow rate we also generated small sucrose spheres with diameters < 40 nm, see Figure 4 b. Decreasing the laser intensity and setting boundaries in the data analysis can be used to neglect those particles. In our experiment, we used similar flow rates as for the DMA measurements.

VI. DATA ANALYSIS

The collected light-scattering images were analyzed using a centroiding algorithm based on Hessian Blob-finding [6]. The blob-center positions at $z = 2.61$ mm are shown in a 2D histogram in Figure 6 a. Due to an imperfect laser beam profile we detected particles outside the laser focus region, which were removed from the dataset by restricting the analysis to the region of the laser path, i. e., the red box in Figure 6 a. In addition to the blob-center position the algorithm provided the integrated intensity of the blob. A resulting intensity distribution is shown in Figure 6 b, showing the same shape as the DMA data in Figure 4 a. To analyze the focusing of 27 nm AuNPs we restricted

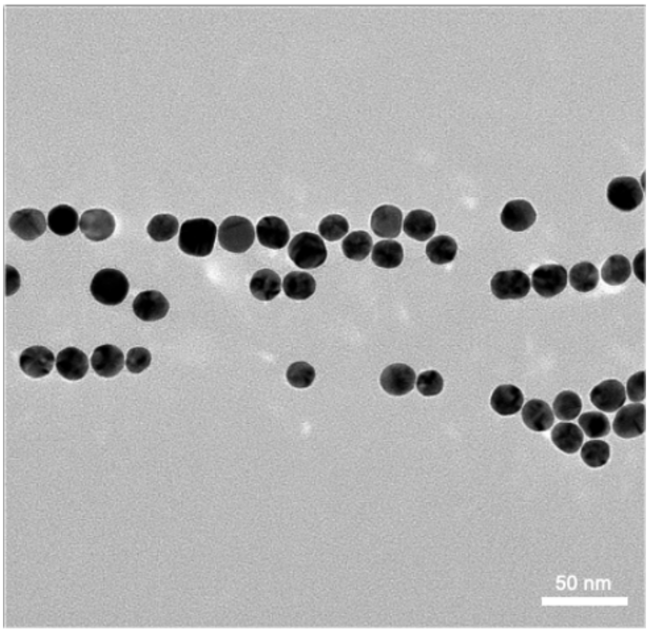

FIG. S5. TEM image of AuNPs after ligand-exchange in 2.5 mM ammonium acetate. The core-size ranged from 18 to 26 nm with an average of 24 nm. The scale bar is 50 nm.

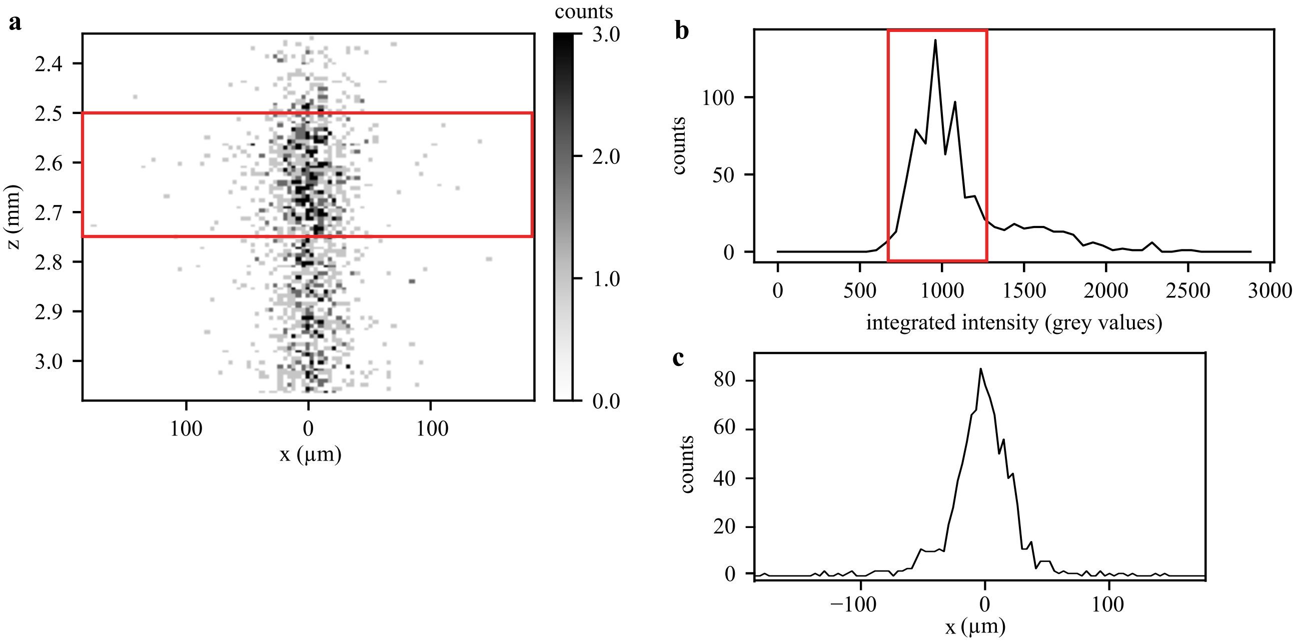

FIG. S6. (a) 2D histogram of the found particle blob center positions at $z = 2.61$ mm distance from the ALS exit of AuNPs. The red marked region is the actual region of the laser focus, which limits the analysis to particles in this region. (b) Integrated intensity distribution of the particle blobs. It shows the same pattern as the DMA data, see Figure 4 a. We limit the analysis to the first peak, corresponding to AuNPs with a diameter of (27±2.25) nm. (c) Projection of the sorted particle positions onto the x-axis. From this distribution, we determine the beam diameter as d_{70}.

our further analysis to particles with an integrated intensity in the red marked region in Figure 6 b. The remaining centroids are projected onto the laser propagation axis (x-axis), as shown in Figure 6 c. From this distribution, we determine the beam diameter as d_{70}.

REFERENCES

[1] R. Neutze, R. Wouts, D. van der Spoel, E. Weckert, and J. Hajdu, Potential for biomolecular imaging with femtosecond x-ray pulses, Nature **406**, 752 (2000).

[2] D. A. Horke, N. Roth, L. Worbs, and J. Küpper, Characterizing gas flow from aerosol particle injectors, J. Appl. Phys. **121**, 123106 (2017), arXiv:1609.09020 [physics].

[3] X. Wang and P. H. McMurry, A design tool for aerodynamic lens systems, Aerosol Sci. Technol. **40**, 320 (2006).

[4] P. Liu, P. J. Ziemann, D. B. Kittelson, and P. H. McMurry, Generating particle beams of controlled dimensions and divergence: I. theory of particle motion in aerodynamic lenses and nozzle expansions, Aerosol Sci. Techn. **22**, 293 (1995).

[5] M. Yon, C. Pibourret, J.-D. Marty, and D. Ciuculescu-Pradines, Easy colorimetric detection of gadolinium ions based on gold nanoparticles: key role of phosphine-sulfonate ligands, Nanoscale Adv. **2**, 4671 (2020).

[6] B. P. Marsh, N. Chada, R. R. Sanganna Gari, K. P. Sigdel, and G. M. King, The Hessian blob algorithm: Precise particle detection in atomic force microscopy imagery, Sci. Rep. **8**, 978 (2018).
